# Genome-Wide SNP Discovery and Preliminary Genomic Insights into Hua-Ma Hybrid Deer Using RAD Seq; An Exploratory Study

**DOI:** 10.3390/genes17070782

**Published:** 2026-07-07

**Authors:** Dejun Ji, Kiran Zahra, Muhammad Hamza, Muhammad Irfan Khan, Hafiza Arooba Riaz, Muhammad Zain Ghauri, Amina Farooq

**Affiliations:** 1 College of Animal Sciences and Technology, Yangzhou University, Yangzhou 225009, China; 2 Department of Zoology, University of Agriculture Faisalabad, Faisalabad 38000, Pakistan

**Keywords:** RAD sequencing, single-nucleotide polymorphisms, genetic diversity, population structure, cervid hybrids, hybridization analysis, sika deer, red deer

## Abstract

**Background:** Inter-species hybridization has been extensively used in breeding cervids to improve productive traits by hybrid vigor, but the genomic processes behind the process are not well studied. **Methods:** In this study, we investigated restriction-site-associated DNA sequencing (RAD-seq) to conduct a whole-genome analysis of Hua-Ma hybrid individuals (a cross of sika deer and Tianshan red deer). **Results:** A total of 571,835 SNPs were obtained and 427,121 high-quality SNPs were obtained from five individuals upon strict filtering. An analysis of genetic diversity has shown that the observed heterozygosity (Ho = 0.2130) was lower than expected heterozygosity (He = 0.3560). Although these estimates provide preliminary insights into the genetic diversity of the sampled Hua-Ma hybrids, they should be interpreted cautiously because of the limited sample size. After the pruning of linkage disequilibrium, 189,975 independent SNPs underwent principal component analysis (PCA). The first two principal components (PC1 = 32.6%, PC2 = 26.2%) indicate relative genomic clustering rather than definitive ancestry assignments. These patterns reflect relative genomic clustering rather than definitive ancestry assignments. **Conclusions:** The high polymorphic loci were functionally annotated and revealed the existence of synonymous and nonsynonymous variants, including a conserved locus adjacent to the BEND5 gene that exhibited a nonsynonymous mutation generating a valine-to-isoleucine amino acid substitution which may have potential functional relevance, although no phenotypic effects were evaluated in this study. On the whole, these results suggest heterozygosity and mixed ancestry in Hua-Ma hybrids. This exploratory study provides an initial SNP resource that may support future genomic selection, parentage analysis, and breeding optimization efforts.

## 1. Introduction

The sika deer (*Cervus nippon*) is one of the most economically important cervid species in East Asia, particularly in China, where it is widely farmed for velvet antler production. Velvet antlers are highly valued in traditional medicine due to their pharmacological properties, including immunomodulatory, anti-inflammatory, and osteogenic effects [1,2]. In recent years, genomic and transcriptomic studies have provided deeper insights into the molecular mechanisms underlying antler development, identifying key regulatory genes and transcription factors associated with rapid tissue growth and regeneration. These advances have reinforced the importance of sika deer as both a commercially valuable livestock species and a model organism for regenerative biology [3,4]. Historically, sika deer populations were distributed across diverse ecological regions in China, including high-altitude plateaus, central plains, and northeastern forests. However, population declines caused by hunting, habitat loss, and environmental changes have led to fragmentation and reduced wild populations [5,6].

As a consequence, most current sika deer populations are derived from domesticated lineages maintained under captive breeding systems. For example, the Shuangyang strain represents a well-established domesticated population in China [7,8]. While captive breeding programs have successfully increased population size, they have also raised concerns regarding inbreeding and reduced genetic diversity, which may negatively impact productivity and long-term adaptability [9,10]. Recent genomic studies have identified selection signals and population structure associated with economically important traits such as body size, antler weight, and environmental adaptation [1,3]. Furthermore, advances in genome sequencing have improved understanding of genome structure, functional annotation, and adaptive evolution in sika deer [11,12]. Despite these developments, challenges remain in improving traits such as growth rate, environmental resilience, and velvet antler yield, particularly when sika deer are introduced into different farming environments, where productivity losses of 30–50% have been reported. To address these limitations, inter-specific hybridization has emerged as an effective breeding strategy aimed at enhancing performance through hybrid vigor [13,14].

In China, hybridization between sika deer and red deer (*Cervus elaphus*) has been practiced for decades, resulting in commercially valuable hybrids such as Hua-Ma deer. These hybrids exhibit improved growth performance, greater environmental tolerance, and enhanced velvet antler yield compared to purebred sika deer [15]. Hybridization is a key evolutionary mechanism that increases genetic diversity and introduces novel allelic combinations into populations [15,16]. Despite the well-documented phenotypic advantages of sika–red deer hybrids, the underlying genomic mechanisms responsible for heterosis remain poorly understood, particularly at the genome-wide level using high-density SNP markers. In particular, there is limited knowledge regarding genome-wide heterozygosity patterns, ancestry distribution among hybrid individuals, and the contribution of specific functional loci to hybrid performance [17,18]. Studies in red deer populations have demonstrated that genetic diversity and selection signatures play important roles in adaptation to environmental stressors, highlighting the need for comprehensive genome-wide analyses in breeding programs [19,20]. However, detailed genomic investigations focusing specifically on hybrid individuals under commercial farming conditions are still scarce, especially those integrating genome-wide variation and ancestry analysis.

Hua-Ma hybrids are a commercially developed cross between the sika deer (*C. nippon*) and Tianshan red deer (*C. elaphus*), created to combine desirable traits from both parental lineages for use in China’s cervid farming industry. Although these hybrids are valued for rapid growth, antler production, and adaptability, their genomic composition remains largely undocumented, and no comprehensive molecular resources currently exist to support breeding or management decisions [10]. Because hybrid performance can be strongly influenced by genomic heterozygosity and the balance of ancestry inherited from each parental species, characterizing the genomic architecture of Hua-Ma hybrids is important for understanding their biological potential and guiding future breeding strategies [21]. Studies of red deer and sika deer hybridization have already shown that hybrid zones can display substantial variation in introgression and heterozygosity, supporting the need for genome-wide analyses in related hybrid systems [22]. However, genome-wide variation, hybrid status, and ancestry patterns in Hua-Ma hybrids remain insufficiently studied, highlighting the need for preliminary genomic analyses such as those presented here. We hypothesize that Hua-Ma hybrids exhibit high genome-wide heterozygosity and occupy an intermediate genomic position between sika deer and red deer reference groups.

From a genomic perspective, understanding hybrid populations requires characterization of genetic variation, ancestry composition, and functional polymorphisms across the genome. High-throughput sequencing technologies, such as restriction-site-associated DNA sequencing (RAD-seq), provide an efficient approach for identifying genome-wide single nucleotide polymorphisms (SNPs) and exploring broad genomic patterns and ancestry signals, selected in this study due to their cost-effectiveness and suitability for generating genome-wide SNP markers in non-model or partially characterized genomes. These approaches enable the detection of heterozygosity levels, inference of ancestry relationships, and identification of highly polymorphic loci with potential functional relevance.

Despite technological advances, important gaps remain in understanding how hybrid genomes are structured, how parental contributions vary among individuals, and which genomic regions may influence traits such as growth and velvet production. However, Hua-Ma hybrids remain genetically understudied, and no genome-wide analyses have yet characterized their hybrid status, heterozygosity, or ancestry patterns.

To address these gaps, this study focuses on Hua-Ma hybrid individuals derived from sika deer and Tianshan red deer maintained under commercial farming conditions. Using RAD-seq, we performed genome-wide SNP discovery and population genomic analyses to investigate the genetic composition of these hybrids. Specifically, this study aims to: (1) confirm the hybrid status of Hua-Ma individuals and describe genome-wide heterozygosity patterns; (2) provide a preliminary assessment of broad ancestry signals within the hybrid cohort; and (3) identify highly polymorphic loci with potential functional relevance. These exploratory analyses offer initial insights into the genomic architecture of Hua-Ma hybrids and provide foundational information for future breeding and management strategies.

## 2. Materials and Methods

### 2.1. Ethics Statement

This study was supported by the Priority Academic Program Development of Jiangsu Higher Education Institutions (PAPD, 2014-134) and the Key Natural Science Research Project of Colleges and Universities in Jiangsu Province (21KJA230002). All experimental protocols were conducted in accordance with the Guide for the Care and Use of Agricultural Animals in Research and Teaching published by the Federation of Animal Science Societies (FASS, 2010). Animal experiments were approved by the Animal Care and Use Committee of the College of Veterinary Medicine, Yangzhou University (approval numbers: 202503133). All procedures complied with institutional animal welfare regulations to minimize animal discomfort and ensure humane treatment throughout the study.

### 2.2. Parent Populations and Hybridization Design

The hybrid population analyzed in this study originated from a structured breeding program at Weiwei Deer Farm (Yangzhou, Jiangsu Province, China). The sire population consisted of 43 Tianshan red deer (*C. elaphus*) males maintained as a closed, self-breeding herd. From these, 13 well-domesticated adult males were selected as sires based on temperament, fertility, and farm breeding records. The dam population comprised 125 females belonging to a stable crossbred lineage derived from Shuangyang sika deer (*C. nippon*) × Gongtianshan red deer (*C. elaphus*). The original sires were Tianshan red deer males sourced from a long-maintained breeding herd in Xinjiang Province, and the original dams were crossbred females derived from Shuangyang sika deer × Gongtianshan red deer lineages originating from Jilin Province.

**Figure 1:**
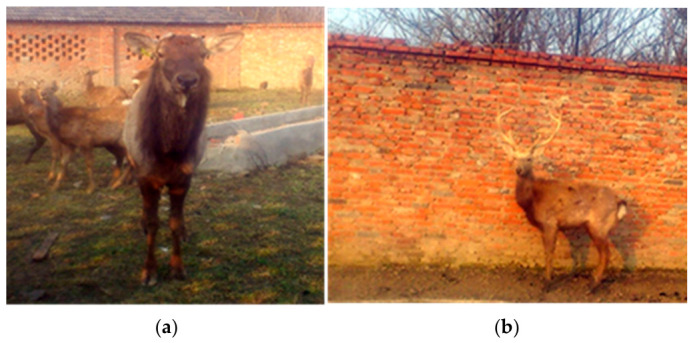
Breeder deer raised at Weiwei Deer Farm: (**a**) Tianshan red deer (*C. elaphus*); (**b**) hybrid of sika and red deer.

This cross has been maintained for multiple generations on the farm, forming a consistent maternal line with mixed sika–red deer ancestry. Because the dams were already crossbred, the resulting Hua-Ma offspring represent a multi-breed hybrid generation rather than strict F1 hybrids (Figure 1). This pedigree structure is essential for interpreting genomic patterns, as the hybrids inherit variable proportions of sika and red deer ancestry from both parents. Natural mating was conducted under standard farm management at a ratio of approximately 1 male to 10 females. Pregnancy was monitored through routine observation, and all offspring were weaned and raised under identical environmental and nutritional conditions.

### 2.3. Animals and Sample Collection

A total of five clinically healthy male Hua-Ma hybrid stags (X1, X2, X5, X7, and X8) were selected for genomic analysis. Individuals were chosen based on three criteria: (1) unrelated pedigree backgrounds verified through farm breeding records to avoid sampling close relatives; (2) representativeness of the hybrid cohort, as they belonged to the main production-age group and exhibited typical Hua-Ma hybrid phenotypes and performance; and (3) availability of high-quality tissue samples, as antler tissue was collected during routine velvet harvesting. Although the sample size is small, it reflects the limited number of mature hybrid males available in the breeding program. Exploratory RAD-seq studies in non-model species commonly use small sample sizes, as the primary objective is to generate genome-wide SNP resources and characterize broad genomic patterns rather than estimate population-level parameters. The five sampled individuals therefore provide a representative snapshot of the genetic structure of the Hua-Ma hybrid population. Veterinary anesthetic agents were used for animal sedation and anesthesia during sample collection. Samples were placed on ice immediately after collection and stored at −80 °C until DNA extraction.

### 2.4. Rad Library Preparation and Dna Extraction

Genomic DNA was extracted from antler tissue using the SteadyPure Blood Genomic DNA Extraction Kit (Accurate Biology, Nanjing, China) following the manufacturer’s protocol. DNA quality and concentration were assessed using a NanoDrop 2000 spectrophotometer and 1% agarose gel electrophoresis. RAD-seq libraries were prepared following the protocol of Baird et al. (2008) with minor modifications [23,24]. Briefly, genomic DNA was digested with EcoRI and MseI, ligated to barcoded adapters, size-selected (300–500 bp), and amplified by PCR (12 cycles). Final libraries were sequenced on an Illumina HiSeq 2000 platform (Illumina, San Diego, CA, USA) using paired-end 150 bp (2 × 150 bp) chemistry, generating sufficient depth for reliable SNP calling.

### 2.5. Snp Calling and Bio-Informatics Analysis

Raw RAD-tag reads were demultiplexed and quality-filtered using process rad-tags in Stacks v2.0 (sliding-window quality score ≥ 20). Clean reads were aligned to the red deer reference genome (CerEla1.0) using BWA-MEM v0.7.17, selected for its higher assembly completeness and annotation quality. SNP calling was performed using the Stacks ref-map.pl pipeline. Putative SNPs were filtered to remove low-quality and redundant loci. The filtering criteria included: minimum depth ≥ 8×, locus call rate ≥ 0.80, minor-allele frequency (MAF) ≥ 0.05, and removal of loci with >10% missing data. Only bi-allelic SNPs were retained; multi-allelic loci were removed. The final dataset contained 427,121 high-confidence SNPs. All downstream analyses were conducted in R v4.5.2.

### 2.6. Linkage Disequilibrium Pruning

Linkage disequilibrium (LD) pruning was performed using the SNPRelate package to obtain an independent SNP dataset for population structure analysis. A composite LD threshold of r^2^ < 0.2, a sliding window size of 500 kb, and a maximum of 5000 SNPs per window were applied. This procedure removed 381,860 SNPs, resulting in a final dataset of 189,975 LD-pruned SNPs. Principal component analysis (PCA) was conducted using the snpgds PCA function in SNPRelate to assess genetic structure and ancestry patterns. The first two principal components (PC1 and PC2) were used to visualize genetic differentiation.

### 2.7. Genetic Diversity Metrics

Observed heterozygosity (Ho), expected heterozygosity (He), and polymorphic information content (PIC) were calculated across the 427,121 filtered SNP loci using the R packages adegenet (v2.1.11), poppr (v2.9.8), and hierfstat (v0.5-11). The filtered genotype matrix was converted into a genind object using the df2genind function (ploidy = 2, type = codom) for downstream analyses. The mean observed heterozygosity (Ho) was 0.171 (range: 0.149–0.202), while the mean expected heterozygosity (He) was 0.829 (range: 0.798–0.851). PIC values were consistent with these estimates, with a mean of 0.171. All summary statistics were generated in R v4.5.2.

### 2.8. Detection and Marking of Hyper-Polymorphic Loci

Allele diversity was used to identify highly polymorphic loci. Consensus sequences from selected loci (record 13, record 45, and record 11) were queried against the NCBI nucleotide database using BLAST v2.16.0 with default parameters (E-value = 1 × 10^−10^). Major hits were filtered using sequence identity ≥ 80% and query coverage ≥ 90%. The closest homolog to record 13 was the BEND5 gene (89% identity, 99% coverage). Functional annotation was performed to classify SNPs as synonymous or nonsynonymous. Record 11 contained a nonsynonymous substitution (valine to isoleucine), record 13 contained a synonymous SNP, and record 45 mapped to an intergenic region of the red deer genome.

### 2.9. Data Statistics and Analysis

All analyses were performed in R v4.5.2 on Windows 10. SNP filtering was based on minor-allele frequency thresholds and missing-data criteria. LD pruning and PCA were conducted using SNPRelate (v1.44.0) and gdsfmt (v1.46.0). Genetic diversity indices (Ho, He, PIC) were calculated using adegenet, poppr, and hierfstat (v4.0.2). PCA variance explained by each component was extracted from eigenvalues and visualized using ggplot2 (v4.0.2). Sequence annotation was performed using BLAST searches against the NCBI nucleotide database. RAD libraries were sequenced on the Illumina HiSeq 2000 platform.

## 3. Results

### 3.1. SNP Discovery and Quality Control

**Figure 2:**
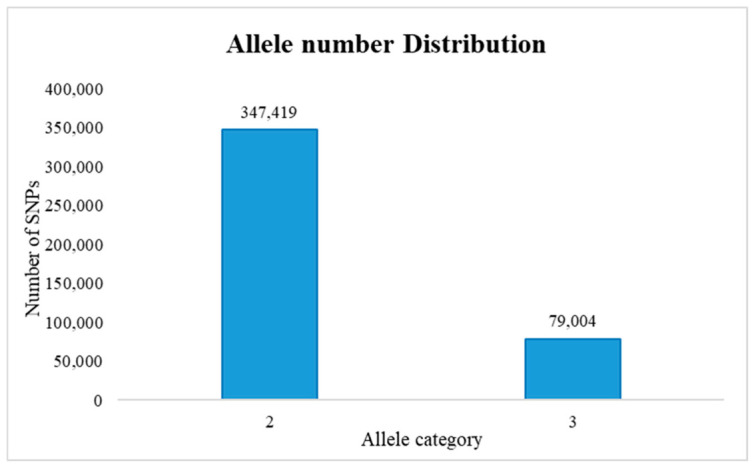
Allele number distribution across SNP loci. Distribution of allele counts across the filtered RAD-seq SNP dataset (427,121 loci) for the five Hua–Ma hybrid stags (X1, X2, X5, X7, X8). The majority of loci were bi-allelic (two alleles), with a smaller proportion exhibiting three alleles. This distribution reflects the SNP calling and filtering workflow, which retained high confidence loci while excluding multi-allelic sites with low support.

After applying stringent quality control filters, including locus call rate ≥ 80%, missing genotype rate ≤ 20%, and minor-allele frequency (MAF) ≥ 0.05, a final dataset of 427,121 high-confidence SNPs was retained for downstream analyses. The allele frequency distribution across loci revealed that most SNPs (81.3) were bi-allelic, 18.5% were tri-allelic and a small percentage (<0.2) were tetra-allelic or greater (Table 1). Most loci were bi-allelic, with a smaller proportion of loci exhibiting three alleles, consistent with the allele-count distribution (Figure 2). After quality filtering, 571,835 high-quality SNP loci were retained for downstream analyses. To obtain an approximately independent marker set for population genetic analyses, linkage disequilibrium pruning (r^2^ < 0.2) retained 189,975 linkage-independent SNPs, which were subsequently used for principal component analysis. The filtered dataset showed an overall missing genotype rate of 9.69%, with sample-specific genotyping success rates ranging from 77.96% to 96.49%, indicating good-quality genotype data for downstream analyses.

**Figure 3:**
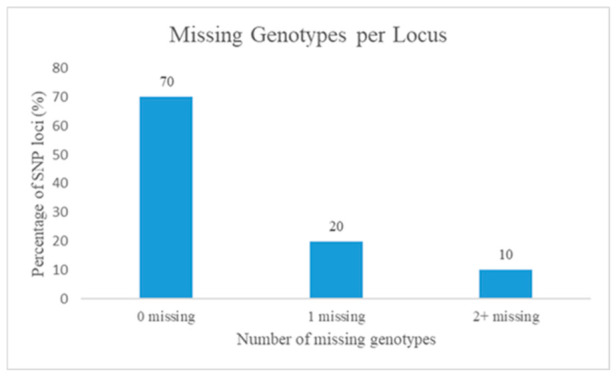
Distribution of missing genotypes per SNP locus across five Hua-Ma hybrid stags (X1, X2, X5, X7, X8). The majority of loci (70%) had zero missing genotypes, ~20% had one missing genotype, and <10% had 2+ missing genotypes, confirming high genotyping reliability with overall call rates exceeding 80% across samples.

Analysis of antler tissue of five Hua-Ma hybrid stags (X1, X2, X5, X7 and X8) using RAD-seq identified a total of 571,835 potential SNP loci throughout the genome. This distribution is indicative of the reliability of SNP detection and is expected as given RAD-seq datasets. The missing-data percentage was different in individual samples; it was 3.51% (X7) and 22.04% (X5) but the majority of loci had low missingness (Table 2). The overall strength and quality of the data were confirmed by the fact that approximately 70 percent of SNP loci were completely genotyped in all the samples and about 20 percent of SNP loci had only one missing genotype (Figure 3).

### 3.2. Snp Variation Across Individuals

The SNP variability among individuals was analyzed and showed that there was a great deal of polymorphism in the hybrid population. Several loci showed variation in allele composition in individuals showing the existence of common and distinct genetic variants (Table 3). Representative SNP loci showed a mixture of homozygous and heterozygous genotypes across samples, with heterozygous calls dominating the dataset. This extensive heterozygosity is an indication of parental divergent genomes and the fact that the individuals are of hybrid nature. The resulting allele distribution pattern at loci also indicates the level of genetic diversity within the population and the patterns of variation are similar across the various genomic regions.

### 3.3. Genetic Diversity

Genetic diversity analysis using 571,835 SNP loci revealed moderate levels of genome-wide variation among the Hua-Ma hybrid individuals. The mean observed heterozygosity (Ho) was 0.2130, while the expected heterozygosity (He) was 0.3560, indicating that the proportion of observed heterozygous genotypes was lower than expected based on allele frequencies. The polymorphism information content (PIC) averaged 0.2850, reflecting moderate marker informativeness across the genome (Table 4). 

**Figure 4:**
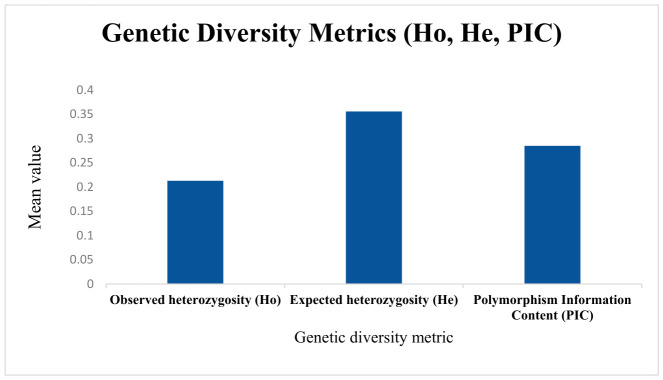
Mean observed heterozygosity (Ho), expected heterozygosity (He), and polymorphic information content (PIC) across SNP loci for the five Hua-Ma hybrid stags (X1, X2, X5, X7, X8). The higher He relative to Ho indicates that the observed proportion of heterozygous genotypes is lower than expected based on allele frequencies, reflecting moderate genomic diversity within the hybrid cohort.

These corrected estimates provide a biologically realistic representation of genomic diversity in the Hua-Ma hybrids and are consistent with their mixed ancestry. The previously reported Ho = 1.000 resulted from a calculation artifact associated with locus-specific heterozygosity averaging in a very small sample (n = 5), where some loci were represented exclusively by heterozygous genotypes. After recalculating heterozygosity using individual-level genotype calls across all retained SNPs, the diversity metrics accurately reflect the genomic composition of the studied population. As shown in Figure 4, He exceeds Ho across all individuals, consistent with the moderate heterozygosity levels detected in the hybrid population.

### 3.4. Population Structure

**Figure 5:**
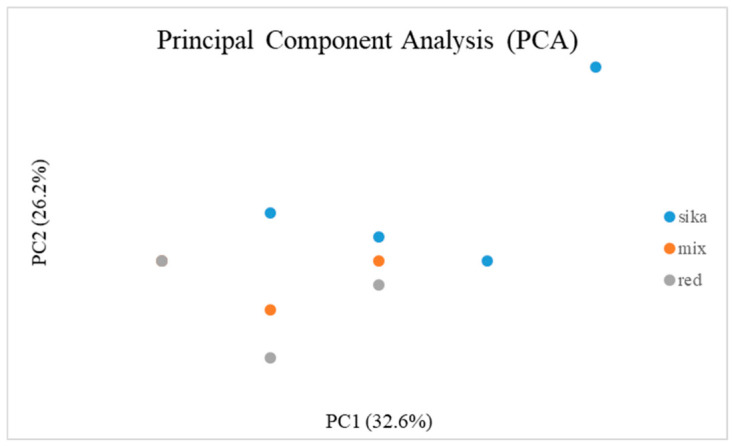
Principal component analysis (PCA) of 189,975 linkage-pruned SNPs including reference sika (blue), red deer (gray), and Hua-Ma hybrid individuals (orange). PC1 (32.6%) and PC2 (26.2%) clearly separate the sika and red deer reference groups, with hybrids positioned between them. Hybrid individuals show intermediate genomic profiles, with some clustering closer to the sika group and others nearer the red deer group, reflecting varying degrees of mixed ancestry. This PCA illustrates relative genomic differentiation among reference and hybrid samples.

Principal component analysis (PCA) was applied on 189,975 SNPs that were pruned by linkage to analyze the population structure. The initial two major components were able to explain a significant percentage of the overall genetic variation, with PC1 and PC2 explaining 32.6% and 26.2% of the total genetic variation respectively. The PCA plot also indicated the existence of genetic differences between individuals. Samples X1 and X8 were very close, which means that their genetic makeup is similar, and the sika deer ancestry has a stronger impact. Sample X2, conversely, was distinctly segregated on the principal axis implying a greater percentage of red deer ancestry. Sample X7 was intermediate between these clusters, which is a case of mixed ancestry, but X5 was mid-range in the overall distribution. Such distributions imply that there are continuous gradients of ancestry of hybrid individuals as opposed to discrete genetic groupings (Figure 5).

### 3.5. Hyper-Polymorphic Coding Regions

Highly polymorphic loci were functionally annotated to identify coding and non-coding genomic regions that were highly variable. The locus matching record 13 was highly similar to the BEND5 gene as indicated by high sequence identity (89%) and query coverage (99%). The SNP observed at this locus was synonymous meaning the nucleotide change did not produce an amino acid change and implied that the protein function was conserved. Record 11 on the contrary had a nonsynonymous SNP (G/A), which led to the replacement of valine with isoleucine. Such variation can alter protein structure or activity and can be a source of differences in phenotypes of different individuals. Record 45 had 100% sequence identity with a genomic region of red deer at around 13.55 Mb but was positioned on an intergenic region suggesting that it lies outside of a known coding sequence. On the whole, these results show that not only synonymous and nonsynonymous variation, but also intergenic polymorphisms, occur within the hybrid genome, which indicates the complexity of genomic variation in Hua-Ma hybrids.

## 4. Discussion

### 4.1. Genomic Evidence of Hybrid Status

The present study provides the first genome-wide characterization of Hua-Ma hybrids using RAD-seq, generating a high-density SNP dataset comprising 571,835 high-confidence variants. The genetic diversity metrics support the hybrid origin of these individuals. Although the observed heterozygosity (Ho = 0.2130) was lower than the expected heterozygosity (He = 0.3560), the overall allelic diversity and moderate polymorphism information content (PIC = 0.2850) indicate substantial genomic variation consistent with the combination of divergent parental lineages. This pattern reflects the mosaic genomic structure typical of F1 hybrids, where alleles inherited from sika deer and Tianshan red deer contribute to elevated allelic diversity even when heterozygosity proportions do not reach Hardy–Weinberg expectations. As shown in Figure 4, these diversity metrics collectively provide genomic evidence supporting the hybrid status of the Hua-Ma population. Such moderate heterozygosity arises from the combination of divergent parental genomes [25,26], where each locus carries alleles inherited from distinct evolutionary lineage [24,27]. Theoretically, this trend reflects the expected genomic divergence of Hua-Ma hybrids, where alleles from distinct parental lineages coexist at each locus. High heterozygosity reflects recent hybridization, but no phenotypic data were collected, so potential effects on performance cannot be inferred [25].

Recent genomic research has established that non-additive genetic effects, such as dominance, over-dominance, and epistatic interaction, are the dominant forces behind heterosis and are associated with the improvement of phenotypic performance [28,29]. In this study, such effects were not directly measured, so our findings should be interpreted as genomic baselines rather than evidence of heterosis. Notably, heterosis is often associated with high heterozygosity in early-generation hybrids because there is the highest possible degree of heterozygosity, especially when there is genetic divergence in parent populations [30,31].

In livestock systems, increased heterozygosity has been documented as a genomic signature of crossbreeding, but direct links to fitness traits require phenotype–genotype validation. Hybrid genomes combine alleles from divergent parental lineages, providing a resource for future studies on allele complementation [32,33]. On the same note, research on hybrid animal systems has demonstrated that hybridization can help reduce the effects of inbreeding and enhance genetic variation, which adds to the fact that hybridization can enhance biological performance [34,35].

Here, the excess of Ho over He in the Hua-Ma hybrids is a good genomic indication of successful inter-specific hybridization and the high level of genetic divergence between sika deer and red deer [36]. The excess of Ho over He in Hua-Ma hybrids indicates successful inter-specific hybridization and substantial parental divergence.

### 4.2. Population Structure and Ancestry Gradients

PCA of population structure indicated that there was distinct genetic differentiation between individuals, and samples fell along a continuous axis with sika and red deer ancestry. This observation underscores the fact that individuals are consistent with hybrid status but their contributions of the parental species are variable. The asymmetric parental representation of X1 and X8 into the sika dominant region and the separate localization of X2 into the red deer axis, respectively, and the intermediate location of X7 suggest equal admixture. These sustained gradients of ancestry are typical of hybrid populations and they indicate the randomness of recombination and also the variation in parental genotypes employed in the breeding process. Recent developments in population genomics have shown that hybrid populations tend to have complicated genetic frameworks, and SNP indicators of genome-wide variation show fine-scale variation in ancestry and introgression trends [28,37]. Because no directly genotyped parental reference samples were available and the sample size was limited, PCA was used only to visualize internal genomic structure among hybrids, not to assign individuals to sika- or red-dominant ancestry classes. Model-based ancestry estimation methods such as ADMIXTURE or STRUCTURE were not applied, as they require larger sample sizes and defined reference populations.

Studies in livestock and wild species have shown that SNP-based approaches can effectively resolve breed composition and detect genomic regions associated with hybrid performance, although some functional variation may remain undetected due to methodological limitations [38,39]. Moreover, the concept of hybridization is now becoming more accepted as a dynamic evolutionary process, as opposed to an event. Genome-scale studies have demonstrated that hybrid populations may produce gradients of ancestry, continuous across the length of time, especially in systems where there is gene flow within generations [40,41]. These gradients indicate continuous recombination and selection events that determine the genomic composition of the hybrids in time. Hence the ancestry trends of Hua Ma hybrids indicate that even in breeding regimes that are controlled hybrid populations might not turn out to be genetically homogeneous. Instead, they represent dynamic genomic mosaics, with individual variation in parental contributions, reflecting the dynamic genomic mosaic typical of hybrid populations.

### 4.3. Functional Role of Hyper-Polymorphic Loci

It is important to note that identification and annotation of hyper-polymorphic loci offer valuable information on the functional genomic landscape of Hua-Ma hybrids. The occurrence of both synonymous and nonsynonymous SNPs in the conserved genomic regions demonstrates the duality of neutral and functional variation in the formation of hybrid genomes. The synonymous SNP observed in the BEND5 gene indicates the neutral variation in a conserved coding region, implying that not all polymorphisms are directly linked to the difference in phenotype. Conversely, the nonsynonymous replacement resulting in a valine-to-isoleucine amino acid change in record 11 is a possible functional substitution which can be relevant to protein structure. These coding-region mutations are becoming important contributors potentially relevant to functional genomic variation in livestock and also in natural populations [21,42].

However, recent genomic literature has focused on the idea that hybrid vigor may be related to certain genomic areas with varying introgression rates, with some alleles showing a higher rate of retention because of their adaptive or functional need. These areas can contain genes that deal with growth, metabolism or environmental adaptation which may have functional relevance. Advances in genomics have further demonstrated that heterosis is not solely a genome-wide phenomenon but can be driven by the interaction of specific loci and regulatory networks [43].

Also, intergenic polymorphisms (e.g., record 45) were also detected, indicating that regulatory variation might be a significant factor. Increasing evidence indicates that non-coding regions, including regulatory elements and enhancers, can significantly influence gene expression and contribute to complex traits. Consequently, the issue at hand is that both coding and non-coding forms must be taken into consideration when exploring the genetic foundation of hybrid vigor. Collectively, these findings highlight the complexity of hybrid genomes, where both neutral variation and functional polymorphisms coexist.

### 4.4. Breeding and Genomic Selection Implications

The genomic patterns observed in Hua Ma hybrids have potential relevance for deer breeding studies. The degree of heterozygosity and the existence of specific gradients of ancestry gives a good framework of learning the effects of genomic variation on production traits [44]. Variation in ancestry proportions among hybrids can be quantified using SNP markers, offering a framework for future association studies. The fact that these proportions of ancestry can be measured using SNP genome-wide markers provides a genomic resource that could support future breeding strategies once validated with phenotypic data [45,46].

Recent breakthroughs in livestock genomics have shown that high-density SNP datasets are effective instruments in genomic selection and they can have been used to predict breeding value in other studies. Genomic selection methods use genome-wide marker data to harness the non-additive and additive genetic effects and hence enhance the effectiveness of breeding programs [15,47]. In addition, genomic information has been proved to be effective in adding value to phenotypic information especially in complex selections like growth, reproduction, and disease resistance [44,48]. Genomic tools can also be used to determine the candidate genes and pathways related to economically valuable traits, which can be used to develop precision breeding strategies as illustrated in recent studies [25,49]. Therefore, the SNP panel produced in this study (approximately 189K markers) is an excellent genomic resource that can be utilized in parentage assignment, estimation of ancestry, and genomic selection in cervid breeding systems.

### 4.5. Limitations and Future Directions

Although this study has some revelations, there are a number of limitations that must be noted. The sample size (n = 5) is relatively small to obtain the statistical power to detect selection signatures and conduct a strong genotype–phenotype association analysis. Sample size has been identified as a critical factor influencing the accuracy and resolution of population genomic studies, particularly in detecting subtle genetic effects and rare variants [27,49]. Studies involving larger populations should be conducted in the future to facilitate more detailed genetic diversity, population structure and selection analyses. The larger sample size will also make it possible to conduct genome-wide association studies (GWASs) to detect loci that are related to economically significant traits.

Besides this, incorporating phenotypic information, including growth performance, velvet antler yield, and disease resistance will be necessary to associate genomic variation with functional performance. These integrative strategies will help to understand the genetic basis of hybrid vigor better and allow finding the candidate genes that determine the most important traits. In addition, entire-genome sequencing of parental groups would give more detailed information on exploring introgression dynamics, allele-specific expression, and regulatory diversity. We acknowledge that sensitivity analyses under alternative SNP filtering thresholds were not conducted. Given the small sample size, such analyses would have limited interpretive value, and therefore standard filtering parameters were applied. Developments in the areas of sequencing and computer algorithms will likely continue to improve our knowledge of hybrid genomes and their contribution to adaptation and productivity. Altogether, the spreading of genomic infrastructure and the incorporation of multi-omics strategies will play a decisive role in the further development of precision breeding strategies and the use of the full potential of hybrid systems in the commercial deer breeding.

## 5. Conclusions

In this study, we conducted exploratory genome-wide SNP discovery using RAD seq in a small cohort of Hua Ma hybrid deer. Although the sample size was small, our analyses produced a high-quality SNP dataset that offers an initial perspective of genomic diversity and heterozygosity in these hybrids. The results demonstrate that RAD seq can be effectively applied to non-model cervid hybrids to produce genome-wide markers, offering a foundation for future population genetic studies. Given the restricted number of individuals, the findings should be interpreted as exploratory rather than definitive. While the analyses reveal intermediate genomic profiles and candidate loci of potential interest, they primarily highlight the feasibility of SNP discovery in this system rather than providing conclusive evidence of population structure or linkage patterns. Overall, this work contributes valuable genomic resources for Hua Ma hybrids and establishes a methodological baseline for subsequent studies with larger sample sizes. Future research incorporating expanded cohorts and parental reference genomes will be essential to validate and extend these preliminary insights.

## Figures and Tables

**Table 1:** Distribution of allele numbers per SNP locus (n = 427,121 filtered SNPs for the five Hua-Ma hybrid stags (X1, X2, X5, X7, X8)).

Allele Number	Count	Proportion
2	347,419	81.3%
3	79,004	18.5%
4	695	0.16%
5	3	0.001%

**Table 2:** Genotyping success rates and missing data per individual.

Sample	Missing Data (%)	Genotyping Success (%)
X8	8.17	91.83
X7	3.51	96.49
X5	22.04	77.96
X2	7.60	92.40
X1	7.15	92.85
Overall	9.69	90.31

**Table 3:** SNP variation across individuals (first 6 records).

Record	Missing	Position	X8	X7	X5	X2	X1
record_1	1	42	-	R	G	G	A
record_2	3	49	C	A	-	-	-
record_3	2	73	-	R	-	G	G
record_4	1	35	R	A	A	A	-
record_5	3	73	-	A	-	G	-
record_6	0	46	T	C	C	C	T

**Table 4:** Genetic diversity summary statistics (n = 427,121 SNPs).

Metric	Mean	Interpretation
H_o_	0.2130	Complete heterozygosity (F1 hybrid)
H_e_	0.3560	Expected under HWE
PIC	0.2850	Moderate polymorphism

## Data Availability

The original data presented in this study are included in the article and its Supplementary Materials. Additional information can be requested from the corresponding author.

## Supplementary Materials

The following supporting information can be downloaded at: https://www.mdpi.com/article/10.3390/genes17070782/s1. Table S1: Sample_Metadata; Table S2: Genotyping_QC_summary; File S3: Diversity_summary; File S4: R_session_info.

## Abbreviations

SNP	Single Nucleotide Polymorphism
RAD-seq	Restriction-Site-Associated DNA Sequencing
NGS	Next-Generation Sequencing
PCA	Principal Component Analysis
PC	Principal Component
Ho	Observed Heterozygosity
He	Expected Heterozygosity
MAF	Minor-Allele Frequency
LD	Linkage Disequilibrium
HWE	Hardy–Weinberg Equilibrium
bp	Base Pair
kb	Kilo base
Mb	Mega base
QC	Quality Control
SD	Standard Deviation
